# Supplementary material for: Ethical and psychosocial considerations for hospital personnel in the Covid-19 crisis: Moral injury and resilience
Source: PLoS One. 2021 Apr 2;16(4):e0249609. doi: 10.1371/journal.pone.0249609 (PMC8018614; doi:10.1371/journal.pone.0249609)
Supplement: S1 File — (DOCX) [file pone.0249609.s002.docx]

Interview Guide: Psychosocial Considerations for Health Care Workers in the COVID-19 pandemic

1. How do you experience the situation in your hospital since the outbreak of COVID-19?
2. Would you like to share with us some examples regarding your experience?

Challenges in hospitals

What challenges do you perceive since the outbreak?

- Stressors
- Work Environment
- Patient care
- Risk perception
- Protective measures
- PPE
- Interaction with colleagues
- Management/leadership

Impact

What impact does working during the pandemic have on hcw?

- Social (Friends/relatives, family, colleagues…)
- Behavioral
- Emotional
- cognitive

Resources/Needs

What resources are of help to support hcw?

What needs do you perceive?

- PSS measures
- Management
- Leadership
- Technical support
- Guidelines
- training

1. Open question: would you like to add something?
