# Supplementary material for: Ethical and psychosocial considerations for hospital personnel in the Covid-19 crisis: Moral injury and resilience
Source: PLoS One. 2021 Apr 2;16(4):e0249609. doi: 10.1371/journal.pone.0249609 (PMC8018614; doi:10.1371/journal.pone.0249609)
Supplement: S2 File — (PDF) [file pone.0249609.s003.pdf]

universität
innsbruck

Büro der Vizerektorin
für Forschung

Innsbruck, May 11, 2020

Certificate of good standing, 18/2020

This document certifies that the

Board for Ethical Questions in Science of the University of Innsbruck

has reviewed the project

"Befragungen zur Befindlichkeit unterschiedlicher Personengruppen in der gegenwärtigen Corona Krise im Rahmen des EU-Projekts NO-FEAR"

of

Univ.-Prof. Dr. Barbara Juen

It is hereby certified that this project is in correspondence with all requirements of the ethical principles and the guidelines of good scientific practice of the University of Innsbruck.

Leopold-Franzens-Universität Innsbruck • Vizerektorin

Univ.-Prof. Dr. Ulrike Tanzer
Vicerector for Research
Universität Innsbruck

Rome, 22/02/2021

Approval by the EERB of the NO-FEAR project of the informed consent for the study "Psychosocial consideration for hospital personnel in the COVID-19 crisis".

To whom it may concern,

On 5 June, 2020 UCSC requested, as partner of the EU Project NO-FEAR (G.A. 786670), the approval by the NO-FEAR EERB of the "Psychosocial consideration for hospital personnel in the COVID-19 crisis" study.

The study was designed and conducted as part of the activities of the NO-FEAR project together with the Universities of Innsbruck and Freiburg (both partners of the project).

The EERB, namely External Ethics Review Board, is an independent group of experts that ensures objectivity, impartiality, and controls conflicts of interest in the framework of the NO-FEAR project. It is composed by three experts in relevant areas to ensure a holistic approach and independence.

UCSC submitted to the EERB the following documents:

- preliminary materials of the article;
- informed consent;
- final annex to technical deliverable (draft);
- NO-FEAR narrative report.

On 10 June, 2020 the three members of the EERB approved the "Psychosocial consideration for hospital personnel in the COVID-19 crisis" study.

Professor Daniele Gui
Principal Investigator of the NO-FEAR project
Department of Surgery
Catholic University of Sacred Heart
