# Supplementary material for: Ethical and psychosocial considerations for hospital personnel in the Covid-19 crisis: Moral injury and resilience
Source: PLoS One. 2021 Apr 2;16(4):e0249609. doi: 10.1371/journal.pone.0249609 (PMC8018614; doi:10.1371/journal.pone.0249609)
Supplement: Attachment — Submitted filename: Response to Reviewers.docx [file pone.0249609.s004.docx]

03.02.2021

Dear Editors,

dear Reviewers,

Thank you for reviewing our submission entitled Ethical and Psychosocial considerations for hospital personnel in the COVID-19 crisis: Moral Injury and Resilience (PONE-D-20-34707).

Attached to this letter we are uploading a marked-up copy of our manuscript that highlights all changes made as well as an unmarked version of the revised paper.

In the following, we want to respond to all points raised by the reviewers.

1. Style requirements

We updated the manuscript according to PLOS ONE’s style requirements.

2. Additional information

We now include our interview guide in the supporting documents section.

Given that we conducted our research in the very beginning of the COVID-19 pandemic, an exploratory approach was necessary to gain insights on the health care worker’s perspective in this new environment. We thus followed the principles of openness and induction, as we stated in the methods section of the revised manuscript. Our interview guide contains topics that provide a framework of orientation to ensure comparability and accompany the communication process. However, instead of asking specific, predetermined types of questions the guide relies on the interaction with the interviewee to steer the interview process and helps constructing questions on the topics of interest as the interview progresses – a method that is widely used in qualitative research (see e.g. Witzel, 2000; Edwards & Holland, 2013, p. 55)

We also now provide the COREQ checklist to ensure that we addressed all points relevant for complete reporting.

3. Data availability statement

We were asked to make all data underlying the findings in our manuscript fully available.

However, in our first submission we pointed out concerns as our interview transcripts contain sensible participant information. Publishing the full transcripts is also excluded in the informed consent our interviewees signed. In order to meet transparency needs, excerpts of the transcripts relevant to the study are made available within the paper.

We also provided an overview of our conceptualized data as in this form it does not contain sensible participant information. It is available in a public repository:

“Full data cannot be shared publicly because they contain potentially identifying or sensible participant information. However, conceptualized data after using grounded theory methodology can be retrieved from doi.org/10.5281/zenodo.4241858”

4. Additional review comments

Finally, Reviewer #2 pointed out concerns regarding our sample size, whether there is a bias in the selection process and whether it is fully representative and statistically significant.

We would like to point out the inherent principles of sampling approaches in qualitative research. We did not use a random sampling approach in order to find a statistically representative sample (as it is indispensable in quantitative research). Instead, we purposefully selected information-rich cases, aiming at in-depth understanding rather than statistically proven generalizations (see e.g. Patton, 2002, p.230). We thus do not view our sample as biased, but consider the selection of highly experienced interviewees directly involved in the response of the pandemic as a quality criterion of our data.

We furthermore believe that our approach is in line with trends of narrative-based medicine in recent years (see e.g. Kalitzkus & Matthiessen, 2009).

Our aim in this manuscript was to develop some basic hypotheses regarding resilience and stress experience of healthcare workers in the first phase of the COVID-19 pandemic. However, we agree that a statistical survey would add to the value of our findings. We are therefore currently conducting survey studies with health care workers. The construction of the surveys is based on the findings from qualitative research presented in this manuscript. Findings from quantitative follow-up studies will be presented at a later stage.

Sources

Edwards, R. & Holland, J. (2013). *What is qualitative interviewing?* Bloomsbury Academic.

Kalitzkus, V. & Matthiessen, P. (2009). Narrative-Based Medicine: Potential, Pitfalls, and Practice. *The Permanente Journal, 13*(1), 80-86.

Patton, M. Q. (2002). *Qualitative research & evaluation methods.* 3rd (ed.). Thousand Oaks, CA: Sage.

Witzel, A. (2000). The Problem-Centered Interview. *Forum: Qualitative Social Research, 1*(1), Art. 22. Available at <https://www.qualitative-research.net/index.php/fqs/article/view/1132/2521>

Thank you for taking our manuscript under further consideration.

Best regards,

Alexander Kreh

Corresponding author
